# microRNA-17 Is the Most Up-Regulated Member of the miR-17-92 Cluster during Early Colon Cancer Evolution

**DOI:** 10.1371/journal.pone.0140503

**Published:** 2015-10-14

**Authors:** Kirsten Nguyen Knudsen, Boye Schnack Nielsen, Jan Lindebjerg, Torben Frøstrup Hansen, René Holst, Flemming Brandt Sørensen

**Affiliations:** 1 Department of Clinical Pathology, Vejle Hospital, Part of Lillebaelt Hospital, Vejle, Denmark; 2 Institute of Regional Health Research, University of Southern Denmark, Odense, Denmark; 3 Bioneer A/S, Hørsholm, Denmark; 4 Department of Oncology, Vejle Hospital, Part of Lillebaelt Hospital, Vejle, Denmark; H.Lee Moffitt Cancer Center & Research Institute, UNITED STATES

## Abstract

Deregulated microRNAs play a role in the development and progression of colon cancer, but little is known about their tissue and cell distribution in the continuum of normal mucosa through the premalignant adenoma to invasive adenocarcinoma. The aim of this study was to examine the expression pattern of the miR-17-92 cluster (miR-17, miR-18, miR-19, miR-20 and miR-92) as well as miR-21, miR-31, miR-135b, and miR-145 in early clinically diagnosed colon cancer. MicroRNAs were analysed by chromogenic *in situ* hybridisation in the normal-adenoma-adenocarcinoma sequence of nine adenocarcinomas developed in mucosal colon polyps. Subsequently, the expression of selected microRNAs was validated in 24 mucosal colon cancer polyps. Expression of miR-17 was confined to the epithelial cells, and the expression levels increased in the transitional zone from normal to adenomatous tissue. The miR-17-92 cluster members, miR-19b, miR-20a, and miR-92a, followed the same expression pattern, but miR-17 was the most predominant. An increased expression of miR-21 was found in the tumour-associated stroma with the most dramatic increase from adenoma to adenocarcinoma, while the number of positive miR-145 fibroblast-like cells in the normal lamina propria (stroma) decreased in a stepwise manner throughout the normal-adenoma-adenocarcinoma sequence. It is concluded that the expression of miR-17, miR-21, and miR-145 changes at early stages of the normal-adenoma-adenocarcinoma sequence. Thus, these microRNAs may play a role in the development of colon cancer.

## Introduction

Colorectal cancer is among one of the most common cancers worldwide[1]. A considerable fraction of these cancers are believed to develop in a stepwise manner from normal colonic mucosa through a premalignant adenoma to the invasive adenocarcinoma as a result of complex genetic and epigenetic changes[2–4]. MicroRNAs (miRNAs) are a class of non-coding RNA mediating post-transcriptional regulation that have been implicated in colorectal carcinogenesis and tumour progression by acting as oncogenes and tumour suppressors[5–7]. The miRNAs, which generally contain ~22 nucleotides, target more than 60% of all protein-coding genes[8], and each miRNA can potentially repress hundreds of target genes[9].

MicroRNAs are expressed as transcripts containing a single miRNA such as miR-21 or a number of mature miRNAs (polycistrons), like the miR-143/145 and miR-17-92 clusters. The miR-17-92 cluster contains six different miRNAs: miR-17, miR-18a, miR-19a, miR-19b, miR-20a, and miR-92a, with a highly similar sequence between miR-19a and miR-19b, and between miR-17 and miR-20a[10]. All cluster members have been found elevated in colorectal cancerous tissue compared to normal tissue, although with varying expression of each individual component[11–13]. Other frequently described up-regulated miRNAs in colorectal cancer are miR-21 and miR-31[14–16], while miR-145 is among the most consistently down-regulated miRNAs[15–18]. Furthermore, miR-135b has been reported to be involved in early tumourogenesis [19,20].

Despite the massive ongoing research on miRNA in colorectal cancer, only a few studies have investigated miRNA changes along the entire normal-adenoma-adenocarcinoma (N-A-AC) sequence[20–24]. Bartley *et al* found a total of 230 differentially expressed miRNAs in the N-A-AC evolutionary model including miR-17, miR-19, miR-92a, and miR-21[21], while other investigators have reported miR-31 and miR-135b to be among the most frequently changed miRNAs[20,22,25]. It has also been shown that miR-21 up-regulation from adenoma to adenocarcinoma is a result of increased expression in cancer-associated stromal fibroblasts in the tumour micro-environment[26]. However, information about the tissue and cell distribution of miRNAs in the continuum of the N-A-AC sequence is still scant. Knowledge of miRNA localisation and expression is of fundamental importance in understanding their exact role in the initiation, development, and progression of colon cancer.

The adenocarcinomas developing in mucosal polyps (ACP) provide the unique opportunity to study the early sequential development of adenocarcinoma within the same patient. Using the ACP of the colon as a model of the N-A-AC sequence, the aim of this study was to describe the expression patterns of the miR-17-92 cluster members as well as miR-21, miR-31, miR-135b, and miR-145 in colon cancer development with focus on their prevalence, tissue distribution, and cellular origin.

## Materials and Methods

The tissue investigated in the present study consisted of two independent sets of clinical, diagnostic specimens: a test set of nine formalin-fixed, paraffin-embedded (FFPE) ACPs and a validation set of 24 FFPE ACPs from the colon. All tissue blocks were obtained from the diagnostic pathology archive of Department of Clinical Pathology, Vejle Hospital. The specimens for the test study were originally diagnosed during a colon cancer screening feasibility study conducted from 2005 through 2006 in the County of Vejle, Denmark, while the validation set originated from referred patients diagnosed at Vejle Hospital from 2005 through 2009. Only conventional adenocarcinomas were included, and mucosal polyps containing serrate adenomas were excluded. Confirmation of diagnosis and re-grading of the adenomatous components in the polyps were accomplished by an experienced gastrointestinal pathologist. Detailed patient information is shown in Table 1.

**Table 1:** Clinico-pathological data for the two study sets.

		Test set	Validation set
		n = 9	n = 24
Variable		No. (%)	No. (%)
Age at surgery, years (mean)		64.7	76.0
	Range	52.3–71.5	48.4–93.6
Gender			
	Female	3 (33%)	9 (37.5%)
	Male	6 (67%)	15 (62.5%)
Tumour location			
	Right	1 (11%)	0 (0%)
	Left	8 (89%)	24 (100%)
TNM stage			
	I	9 (100%)	21 (87.5%)
	II	0 (0%)	0 (0%)
	III	0 (0%)	2 (8.3%)
	IV	0 (0%)	1 (4.2%)^a^
Histology, cancer			
	Adenocarcinoma	9 (100%)	24 (100%)
Histology, adenoma			
	Tubular	4 (44%)	11 (46%)
	Tubulovillous	5 (56%)	11 (46%)
	Villous	0 (0%)	2 (8%)
Vascular invasion		1 (11%)	0 (0%)
Mismatch repair protein			
	Normal expression	9 (100%)	23 (96%)
	Loss	0 (0%)	1 (4%)

^a^This patient had a synchronic T3 colon cancer

At study onset, each histological section contained all three components of the ACPs, *i*.*e*. normal mucosa, adenomatous tissue, and invasive adenocarcinoma. However, as more sections were cut from the tissue blocks some areas of interest were missing, and thus two specimens from the test set and up to six from the validation set failed to give complete data from all three compartments of the N-A-AC sequence.

The study was approved by the Regional Scientific Ethical Committees for Southern Denmark (ID# S-20120075) and granted a waiver of informed consent. The study was registered at the Danish Data Protection Agency, and The Danish Registry of Human Tissue Utilisation was consulted before any tissue samples were used.

### *In situ* hybridisation analysis

*In situ* hybridisation (ISH) for miR-17, miR-21, miR-145, miR-126, miR-31, miR-125b, miR-135b, miR-200b, miR-18a, miR19b, miR-20a, and miR-92a was essentially performed as described previously[27]. The miRNA probe sequences and experimental details are presented in S1 Table. In brief, the ISH analysis was performed on 6 μm thick sections using a Tecan Evo instrument (Männedorf, Switzerland). Assay optimisation to determine optimal probe concentrations and hybridisation temperatures was accomplished prior to the analysis. Sections were pre-digested with proteinase-K (15 μg/ml) at 37°C for 8 minutes, pre-hybridised at 57°C for 15 minutes, and hybridised with double-carboxyfluorescein (FAM) labelled Locked Nucleic Acid (LNA) probes (Exiqon A/S, Denmark). After stringent washes in saline-sodium citrate buffer, the probes were detected with alkaline phosphatase-conjugated sheep anti-FAM Fab fragments followed by incubation in substrate containing 4-nitroblue tetrazolium and 5-bromo-4-chloro-3’-Indolylphosphate (Roche, Denmark) resulting in a dark-blue staining, and finally counterstained with nuclear fast red (Vector Laboratories, CA)

### Morphological evaluation of miRNA expression and β-catenin

All slides were evaluated subjectively and scored semi-quantitatively according to miRNA intensity (0 = negative, 1 = weak, 2 = strong) and the proportion of stained cells (0 = <50%, 1 = >50%) except for miR-21. The total score was determined by adding the intensity and proportion scores and dichotomising the sum into “low” (score 0–1) or “high” (score 2–3) expression. Owing to a broad range of miR-21 positive cells in the samples, the proportion of miR-21 stained cells was categorized as 0 = <1%, 1 = 1%-50% and 2 = >50%, and the intensity score was employed as described above. The total miR-21 score was divided into three categories: 0–1 = low; 2–3 = moderate; 4 = high expression.

β-catenin expression was evaluated for membranous, cytoplasmic, and nuclear staining. Since the cytoplasmic reaction was homogenously distributed in the tumour compartments, only the intensity was scored (weak, moderate, and strong). Because the cytoplasmic staining obscured the membranous immunoreaction in many adenomatous and invasive areas, membranous staining was not scored in these compartments. Nuclear staining was divided into two groups: negative = no nuclear reaction seen and positive = ranging from a few scattered positive cells, focal reaction, or diffuse reaction.

### Image analysis

The morphological evaluation documented a pronounced up-regulation of miR-17 in the epithelial cells, which called for further analysis using the following objective approach: Digital whole slide images were obtained with an x20 objective using an Axio Scan Z1 bright field scanner (Carl Zeiss, Germany). Image analysis of the digital slides was performed using VisiomorphDP software (Visiopharm, Denmark). In the 16 cases, quantitative estimates of the miR-17 ISH signal were obtained in regions with normal colonic mucosa (N), low grade adenoma (LGA), high grade adenoma (HGA), and adenocarcinoma, where such homogenous tissue components were evident. The regions were identified by an experienced gastrointestinal pathologist and encircled as regions of interest (ROI) in the digital whole slides. Eight cases were analysed in duplicate and the data from the two slides were pooled. The average areas evaluated (of ROIs) were: N: 5.2 mm^2^ (n = 24); LGA: 6.1 mm^2^ (n = 9); HGA: 7.4 mm^2^ (n = 21), and adenocarcinoma: 8.2 mm^2^ (n = 24), where n is the number of representative ROIs. A pixel classifier was trained to discriminate the blue ISH signal from the red counterstain, the unstained and weakly stained tissue, and the tissue-free areas, and with an intensity threshold discriminating medium blue from intense blue. The following parameters were obtained during image processing from each ROI: area of medium blue, area of intense blue, and the total area of the individual ROIs. The relative area fractions, areas of medium + intense blue divided with the total area (arbitrary unit), were considered representative for the relative miR-17 expression levels.

### Immunohistochemistry (IHC)

IHC was performed on 4 μm tissue sections from the same blocks used for ISH analysis. EnVision FLEX+, Mouse, High pH, (Link) (Dako, Glostrup, Denmark code K8002) was used for the epitope retrieval and IHC staining.

For phosphatase and tensin homolog (PTEN) analysis, the slides were incubated with PTEN antibody (1:200, Dako, Denmark, code M3627) for 30 minutes, amplified with mouse link antibody for 20 minutes followed by horseradish peroxidase-polymer detection for 30 minutes. Antibody staining was performed on a Dako Autostainer Plus (Dako, Denmark) using 3,3’-diaminobenzidine as chromogen and Mayers hematoxylin as counterstain. Complete negative IHC reaction was considered as loss of PTEN.

Mismatch repair protein status had been performed routinely using IHC on approximately half of the specimens during the primary patho-anatomical diagnostic procedure. The remaining cases were stained with antibodies against MLH1 (1:100, Novocastra, UK, code NCL-L-MLH1), MSH2 (1:100, Novocastra, UK, code NCL-MSH2), MSH6 (BD Transduction Laboratories, USA, code 610919) and PMS2 (1:500, BD Pharmingen, USA, code 556415). Non-neoplastic cells within and around the tumour served as internal positive control, and for negative controls the primary antibody was omitted.

IHC analyses for smooth muscle actin (1:1000, Dako, Denmark, M0851), desmin (1:400, Dako, Denmark, M0760) and h-caldesmon (1:200, Dako, Denmark, M3557) were performed on specimens from the test set, while analysis for β-catenin (1:2000, BD Biosciences, USA, code 610153) was performed on the validation set.

### Statistical analysis

Continuous miR-17 data obtained from the image analysis were log-transformed to yield a normal distribution of residuals. To adjust for within-subject variability, a multivariate mixed effects linear regression model with random effects for specimen ID was utilised to examine correlations between log-miR-17 and the fixed covariates: tissue type, age, gender, and histology of the adenoma. Afterwards, linear combinations of estimators were used to compare miR-17 expression levels between groups. P-values less than 0.05 were considered statistically significant. All analyses were performed in STATA version 13 (STATACorp, TX, USA).

## Results

### Test cases: morphological evaluation

Based on literature reviews[5–7,21,22,28–30], we chose to examine the expression of miR-17, miR-21, miR-31, miR-125b, miR-126, miR-135b, miR-145, and miR-200b in the test group of nine ACPs.

**Fig 1:**
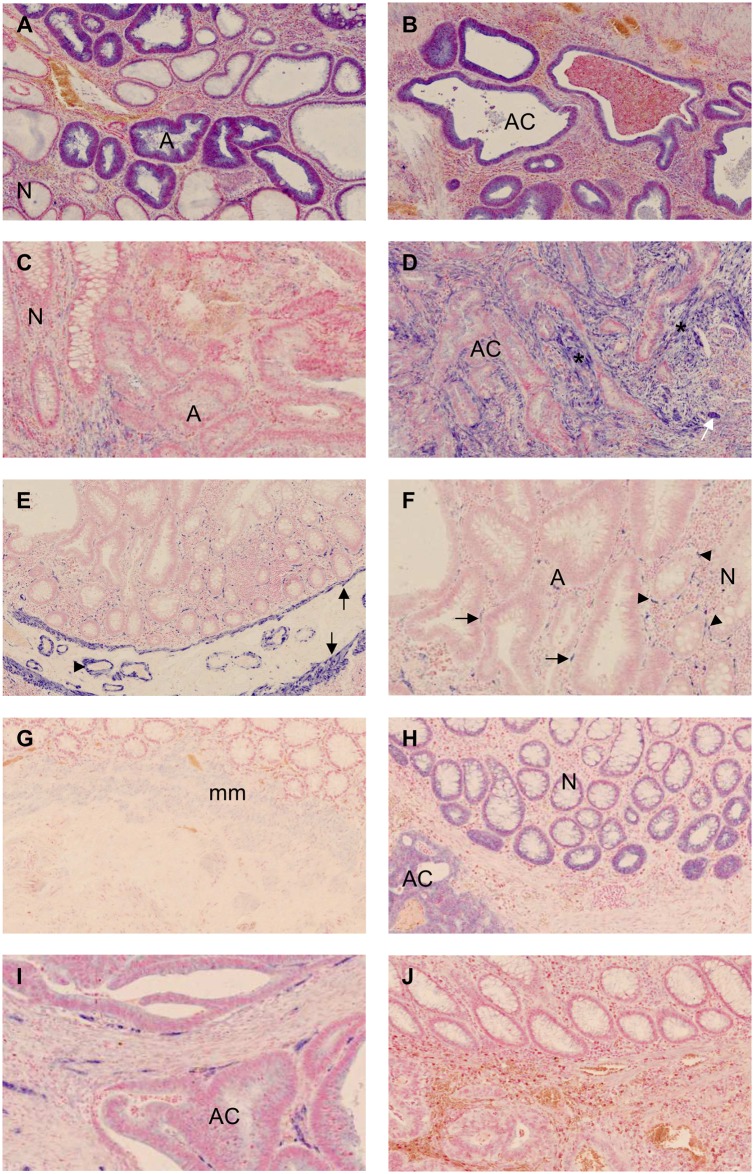
Expression of miR-17, miR-21, miR-145, miR-125, miR-200b, and miR-126 as obtained in the study of the test cases. **(A & B)** miR-17 in normal (N) and adenomatous (A) tissue and in adenocarcinoma (AC); **(C & D)** miR-21 expression in stroma of the adenoma compared to normal tissue and in the cancer-associated stroma cells(*). A small group of positive tumour epithelial cells is also present (white arrow); **(E)** miR-145 is seen in smooth muscle cells (arrow) and vascular smooth muscle cells (arrowhead); in the magnification (**F**) a reduced number of miR-145 positive fibroblast-like cells are found in the adenoma (arrow) compared to normal tissue (arrowhead); **(G)** Faint miR-125b signal was seen in the muscularis mucosa (mm); **(H)** miR-200b was seen in the epithelial cells at base of the crypts, but in this case also in the epithelial cancer cells; **(I)** miR-126 is exclusively seen in the endothelial cells; **(J)** The scramble probe showed only discrete background staining.

Of the eight tested miRNAs, miR-17, miR-21, and miR-145 showed differential expression in the N-A-AC sequence (Fig 1A–1F). In seven out of nine cases, an increased miR-17 expression in the epithelial cells was seen in the transitional zone from normal to adenomatous tissue. The expression was sustained in the cancerous epithelial cells. In all specimens, miR-21 expression was predominantly found in the fibroblast-like stromal cells of the dysplastic and invasive regions of the ACPs with only sparse and focal reaction in the cancerous epithelial cells. The expression pattern seemed to increase in a stepwise manner throughout the N-A-C sequence. All cases showed strong expression of miR-145 seen in the smooth muscle cells (SMC) and vascular smooth muscle cells (VSMC) as well as in fibroblast-like stromal cells. The latter cells were localised in close proximity of the epithelial crypts and appeared to decrease in the adenomatous and cancerous tissue in six out of seven available cases. IHC on the same samples revealed a positive reaction for smooth muscle α-actin and desmin, while h-caldesmon was negative, suggesting that these miR-145-positive cells are of myofibroblastic origin.

A positive signal for miR-125b, miR-126, and miR-200b was present in the specimens, however, no differential expression for any of these three miRs was observed when comparing the three compartments (Fig 1G–1I). A weak miR-125b signal was seen in the fibroblasts in the normal mucosa and SMC in five of nine cases. All specimens showed miR-200b positive epithelial cells, especially at the base of normal colonic crypts, as well as a positive signal in the adenomatous and cancerous epithelium. miR-126 expression was confined to the endothelial cells and was present in all three compartments.

No expression of miR-31 and miR-135b was detected in the nine test specimens.

### Validation study: Morphological evaluation of miR-17, miR-21, and miR-145 expression

Based on the results obtained from the test set, analyses of miR-17, miR-21, and miR-145 were further pursued in the validation set of 24 ACPs. The miR-17 signal was found exclusively in the epithelial cells. Low expression was seen in the normal epithelial cells at the base of the crypts, while high expression was observed in the transitional zone from normal to adenomatous tissue in 96% (23/24) of the cases (Table 2). The expression was sustained in the adenocarcinoma in 96% (22/23) of the cases, while the signal decreased in one case (4%). Only one specimen showed increased expression from adenoma to adenocarcinoma. These two cases did not differ clinically from the other cases of the validation set.

**Table 2:** Results from the validation study of miR-17, miR-21, and miR-145-expression in the normal-adenoma-adenocarcinoma sequence.

	Normal	Adenoma	Adenocarcinoma
**miR-17**			
N	24	24	24
Low	24 (100%)	1 (4%)	1 (4%)
High	0	23 (96%)	23 (96%)
**miR-21**			
N	24	24	22^a^
Low	24 (100%)	7 (29%)	0
Moderate	0	16 (67%)	7 (32%)
High	0	1 (4%)	15 (68%)
**miR-145**			
N	23^a^	22^a^	22^a^
Low	0	11 (50%)	19 (86%)
High	23 (100%)	11 (50%)	3 (14%)

^a^Data missing due to specimens failing to include all three compartments.

Corresponding to the findings in the test set, miR-21 expression was predominantly found in the stromal cells surrounding the dysplastic and cancerous glands. One case, however, showed miR-21 expression in the cancerous epithelial cells, and in three other specimens focal expression in the epithelial tumour cells was also observed. No histopathological or clinical differences were documented among these four cases compared to the other 20 cases. Moderate expression of miR-21 appeared in the transitional zone from normal to adenoma in the stromal cells in 71% (17/24) of the cases. The remaining cases showed increased expression from adenoma to adenocarcinoma (Table 2). In 41% (9/22) of the cases, the up-regulation was intensified throughout the N-A-AC sequence with a moderate miR-21 expression in the adenoma and high expression in the invasive foci.

The analysis of miR-145 showed positive pericryptal fibroblast-like stromal cells in the normal lamina propria in addition to the SMC of arteries and muscle layers in the colon wall. In half of the cases, the number of miR-145 positive pericryptal, fibroblast-like cells decreased in the transitional zone from normal to adenomatous tissue. This decrease was even more evident when comparing normal to cancerous tissue with a reduction seen in 86% (19/22) of the cases (Table 2). The positive signal in the SMC and VSMC remained unchanged throughout all three compartments.

Analysis for miR-135b was repeated in all 24 validation specimens, but no ISH signal was found. The case with mismatch repair protein deficiency did not display a miRNA expression profile different from the proficient cases, neither did the three cases with metastasis.

### Expression of β-catenin in the N-A-AC sequence

Distinct predominant membranous and weak cytoplasmic staining for β-catenin was present in all 24 cases (100%) of the non-neoplastic epithelium, while no nuclear accumulation was seen (S2 Table and A in S1 Fig). In the adenomatous components, cytoplasmic expression was increased in 95% (20/21) of the cases (S2 Table). 62% (13/21) of the adenomas also displayed positive nuclear staining, whereas this was seen in 95% (20/21) of the adenocarcinomas, most often at the invasive front and/or in tumour budding cells (B+C in S1 Fig). Increased cytoplasmic reaction was seen in all 21 (100%) adenocarcinomas (S2 Table).

### Image analysis of miR-17

**Fig 2:**
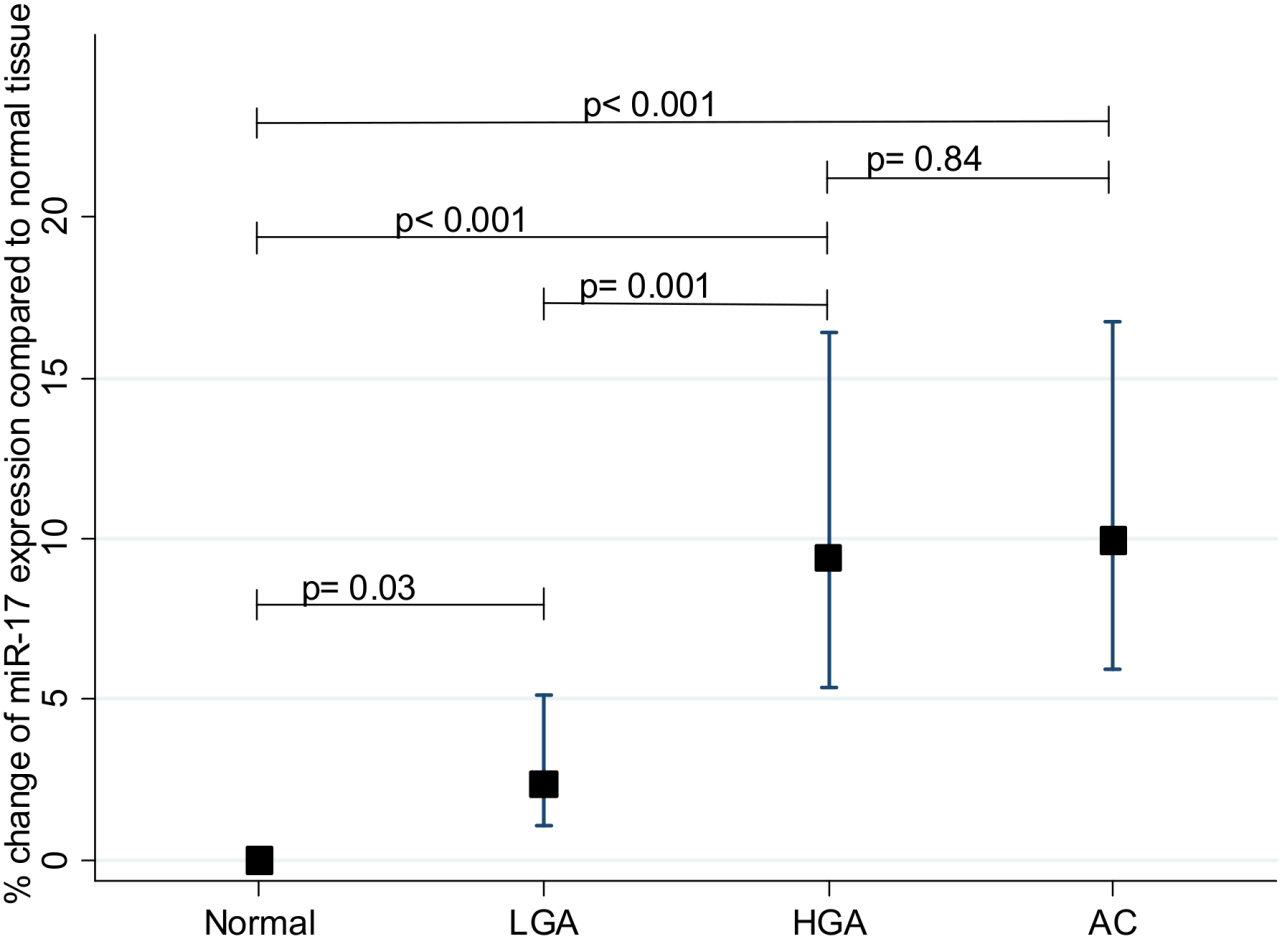
Expression of miR-17 in colon cancer development. Increased expression of miR-17 in low grade adenoma (LGA), high grade adenoma (HGA) and adenocarcinoma (AC) of the colon compared to normal tissue

A subset of 16 randomly chosen specimens from the validation set was further examined by image analysis to obtain quantitative miR-17 expression estimates in areas with normal tissue, LGA, HGA and adenocarcinoma. Estimates of the miR-17 expression were obtained as area fractions (*i*.*e*. stained area divided with the total area) and statistical analyses were performed on log transformed data. Results from the multivariate mixed linear regression are shown in Table 3. Log-miR-17 increased from normal tissue to low and high grade adenomatous tissue and also to invasive cancer (p = 0.03, p<0.001 and p<0.001 respectively; intra-class correlation coefficient = 0.19). Fig 2 shows a total increase of 10% (of the arbitrary unit) throughout the N-A-AC sequence, and that the up-regulation occurred in a stepwise manner starting with a 2.4% rise from normal to LGA (p = 0.03) and an additional 6.9% increase from low grade to HGA (p<0.001). No difference in log-miR-17 expression was seen in the progression from HGA to adenocarcinoma (p = 0.84). The histological type of the adenoma, tubular, villous or tubulo-villous, was not associated to log-miR-17 expression, however, an association with the age of male patients was seen (p<0.001) (Table 3).

**Table 3:** Multivariate mixed effects linear regression of log-miR-17, as obtained by image analysis, in normal, adenomatous and invasive tissue of the colon.

		N	Log-miR-17	P value
			Coefficient (SE)	
Variable				
**Tissue type**				
	Normal	16	ref.	
	Low grade adenoma	5	0.88 (0.41)	**0.03**
	High grade adenoma	13	2.23 (0.28)	**<0.001**
	Adenocarcinoma	16	2.30 (0.27)	**<0.001**
**Adenoma histology**				
	Tubular	16	ref.	
	Tubulovillous	16	-0.01 (0.34)	0.98
	Villous	16	0.78 (0.65)	0.23
**Gender specific age**				
	Female	5	-0.003 (0.03)	0.91
	Male	11	0.09 (0.02)	<0.001

### Expression of miR-17-92 cluster members

**Fig 3:**
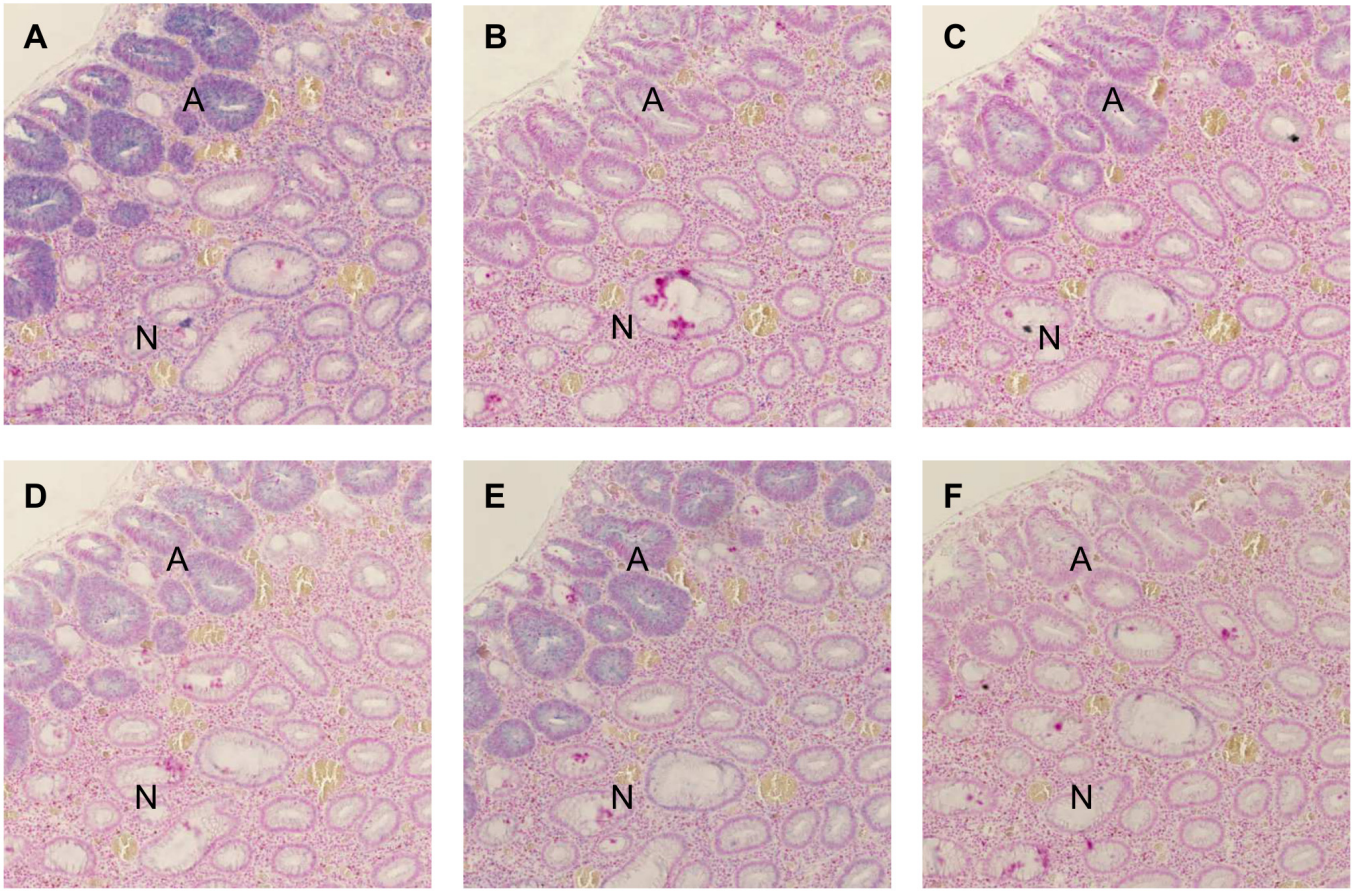
*In situ* hybridisation of the miR17-92 cluster members in normal and adenomatous colonic mucosa. **(A)** Expression of miR-17; **(B)** miR-18a; **(C)** miR-19b; **(D)** miR-20a; **(E)** miR-92a and **(F)** scramble probe in normal (N) and adenomatous (A) tissue. Note the increased staining intensity in the adenomatous crypts compared to the normal crypt.

miR-17 is one of six mature miRNAs encoded from the polycistronic miR-17-92 cluster. Hence, six cases from the validation set with the subjectively strongest miR-17 expression were selected for further ISH analysis regarding cluster members miR-18a, miR-19b, miR-20a, and miR-92a. The miR-19b probe was considered common for the two miR-19 isoforms, as miR-19a differs from miR-19b by only a single nucleotide making 100% discrimination unlikely to occur in the ISH assay. The miR-17 probe sequence differs from miR-20a by three nucleotide positions (S1 Table). The signals of miR-19b, miR-20a, and miR-92a were, like miR-17, confined to the epithelial cells, but the expression was weaker than that seen for miR-17 (Fig 3A and 3C–3E). An up-regulation was observed in the transitional zone from normal to adenomatous tissue, and the up-regulated expression was sustained in the adenocarcinoma. A very faint miR-18a signal in the epithelial cells was seen in four out of six cases in the transitional zone from normal to precancerous tissue (Fig 3B), while more intense ISH signal was found in a few small, rounded lymphocyte-like cells in the stroma of the lamina propria.

### PTEN expression is not related to miR-17 or miR-21 expression

All cases from both study sets were stained for PTEN, a validated target for both miR-17 and miR-21[31,32]. In the validation set, 21% (5/24) of the ACPs showed a complete loss of PTEN but only focally within in the epithelium of the adenomatous and/or invasive areas. However, an inverse association of PTEN and miR-17 expression and miR-21 expression was not observed. No PTEN loss was seen the nine test cases.

## Discussion

In this study, we utilised clinical, diagnostic specimens of colon lesions containing the N-A-AC sequence to explore the expression of a series of miRNAs known from the literature [5–7,28] to be expressed during the development of colon cancer; miR-17, miR-21, miR-31, miR-125b, miR-126, miR-135b, miR-145, and miR-200b. Employing chromogenic ISH, we found that expression of miR-17, miR-21, and miR-145 was particularly dynamic in the early phases of colon cancer development. Image analysis of the miR-17 stained lesions indicated that this miRNA is induced in the early transition from N to LGA and even more dramatically to HGA. The miR-17 expression was found to be of epithelial origin as was also the case for the other miRNAs in the miR-17-92 cluster. miR-17 was found to be the most prevalent miRNA from the miR-17-92 cluster.

Our study is the first to show that miR-17 *in situ* expression increases early from normal mucosa to LGA and reaches a plateau of expression in the HGA which is not exaggerated in manifest adenocarcinoma. This finding is consistent with findings by Bartley *et al*, who reported a similar miR-17 expression pattern using qRT-PCR[21]. Other studies have found that miR-17 levels continued to rise from adenoma to adenocarcinoma[11,33,34], but contrary to our study and the findings by Bartley *et al*, these studies did not compare tissue obtained from the same individuals, and may thus be associated with increased inter-individual variation.

Based on simple, morphological comparison of ISH signal staining intensities, we found miR-17 to be the most up-regulated cluster member in both adenomatous and cancerous tissue, followed by miR-92a, miR-20a, miR-19b, and miR-18a. Humphreys *et al*, using qRT-PCR technique, also found miR-17 to be the highest expressed miR in the miR-17-92 cluster in Dukes A and C colorectal cancers[13], whereas qRT-PCR results from other studies found miR-92a to be the most profoundly expressed miRNA in adenomatous and/or cancerous colorectal tissue[11,12,33]. All these three studies, however, corroborate our finding that miR-18a is the least expressed cluster member, and that miR-19a/b and miR-20a expression are consistently higher than miR-18a[11–13,33]. It cannot be excluded that the binding affinity (melting temperatures) of the LNA probes employed may have contributed to the different staining intensities observed.

We found the expression of all the studied miR-17-92 cluster members to be confined to the epithelial cells. Similar findings of miR-17 and miR-92a expression have been reported by other investigators[31,33–35], although one group also observed some miR-17 positive stromal cells[35]. Interestingly, we observed an intense miR-18a signal in a few small, rounded lymphocyte-like stromal cells in the lamina propria in four of six specimens. However, since no other cluster member was expressed, the signal is most likely to represent cross-hybridisation with another RNA target.

The role of miR-17 in colon epithelial cells in cancer development is still unclear. One possible function could be the inhibition of E2F transcription factor 1, *E2F1*, a putative tumour suppressor gene. Increased expression of E2F1 has been linked to decreased proliferation and increased apoptosis in colorectal cancer[36,37]. Interestingly, an inverse relationship between E2F1 and miR-17 has been shown in colon cancer tissue[35,38]. Monzo *et al* showed that transfection of anti-miR-17 resulted in increased E2F1 expression and decreased cell proliferation[35], and Kanaan *et al* showed that the transfection of miR-17 into HT-29 colorectal adenocarcinoma cells lead to decreased E2F1 expression[39]. In a study on neuronal lineage differentiation of unrestricted somatic stem cells, a direct interaction between miR-17 and E2F1 has been documented[40]. Thus, increased expression of miR-17 in colon epithelial cells could contribute to tumourogenesis by promoting cell proliferation. Additional studies are indeed needed to evaluate the relationship between miR-17 and E2F1 in colorectal cancer and will require careful examination of their respective expression in sufficient morphologically characteristic regions present in such unique samples. The study sets employed in this study did not allow for the additional analysis since the representative regions were no longer available in a substantial fraction of the material.

The putative onco-miR, miR-21, was mainly found in stromal cells surrounding the dysplastic, adenomatous and cancerous epithelial cells. The intensity of miR-21 expression increased throughout the N-A-AC sequence with the highest expression seen in cancer-associated stroma. Our results confirm the findings of Yamamichi *et al*[26]. Except for one case with miR-21 positive tumour epithelium only, a predominantly stromal miR-21 expression was displayed in the remaining 23+9 specimens. Considering the small sample size used in the study by Yamamichi *et al* as well as this present one, it cannot be entirely excluded that early miR-21 expression could be an epithelial phenomenon. Nevertheless, large studies on miR-21 in more advanced colorectal cancers clearly demonstrate miR-21 to be localised in the stromal compartment[27,41,42].

We observed that the number of miR-145 positive, pericryptal fibroblast-like cells was reduced gradually in the N-A-AC sequence. Decreased levels of miR-145 in colorectal cancer compared to normal colon, as measured by qPCR, have been found in numerous studies, and miR-145 has long been considered a possible tumour suppressor. However, recently this theory was challenged[43]. Using ISH, Chivukula *et al* found that miR-145 is expressed only in mesenchymal cells and not epithelial cells in mouse colon, whereas miR-145 was nearly undetectable by qRT-PCR in purified mouse and human intestinal epithelium[44]. The authors concluded that miR-145 functioning as an epithelial tumour suppressor was unlikely, and that previous results of miR-145 loss was more likely related to differences in cellular composition of normal and cancerous tissue[43,44]. Our findings are in agreement with this statement, and the data imply miRNA localisation as an outmost important aspect of research in this field. Consistent with Chivukula *et al*, our ISH results showed that miR-145 is only expressed in cells of mesenchymal origin. In addition, we observed that miR-145 expression changed in the N-A-AC sequence with a reduction of miR-145 positive pericryptal cells of possible, h-caldesmon-negative, myofibroblast origin. Better characterisation of the miR-145 positive cells may help to elucidate their exact origin and clarify whether the miR-145 expression is decreased in these cells, or if the reduction could possible be caused by a loss of the cell population.

Unexpectedly, no miR-135b ISH signal was obtained in any of the 24 validation specimens or in the pilot test specimens. This contrasts with several reports of highly elevated levels in adenomas and adenocarcinomas *vs*. normal tissue as obtained by qRT-PCR technique[19,20,22,45]. A possible explanation could be a low copy number of miR-135b, several hundred-fold lower than that of *e*.*g*. miR-21 in colorectal tissue[46], making detection by ISH difficult due to its low sensitivity compared to PCR. We did not pursue miR-31 in the validation set due to its association with more advanced cancers[47,48]. Moreover, a recent study indicated miR-31 to be involved in the serrated pathway[49], and such lesions were excluded from our material.

Alterations of the adenomatous polyposis coli (APC)/β-catenin pathway is a well-known early genetic event in sporadic colorectal carcinogenesis[50,51] leading to intracellular accumulation of β-catenin. β-catenin may then translocate from the cytoplasm to the nucleus where it modulates a number of important target genes[51]. We therefore evaluated β-catenin expression in our ACPs. Immunohistochemical staining showed increased cytoplasmic accumulation and nuclear expression of β-catenin in the transition from normal mucosa to adenoma, and further on to adenocarcinoma similarly to other studies[52–54] reaffirming the early carcinogenic process.

*PTEN* is a tumour suppressor gene involved in cellular regulatory functions such as cell cycle arrest and apoptosis through inhibition of the PIK3 pathway[55]. PTEN protein levels decrease throughout the N-A-AC sequence[56], and miR-17 and miR-21 are among the reported inhibitors[31,32,57]. Surprisingly, no obvious inverse relationship between these miRNAs and PTEN was found in this study. Both Fang *et al* and Xiong *et al* applied a more extensive immunoscore system in their PTEN evaluation and found an inverse relationship between PTEN and miR-17 and miR-21, respectively[31,32]. Most likely, the failure to discover such a relationship in our study can be related to the limited number of cases investigated. Fang *et al* found an inverse relationship between miR-17 and PTEN in a large study of 295 patients with colorectal cancer[31]. Aside from the large case number, their study also differed regarding the patient groups examined. While their material consisted mainly of high clinical stage colorectal cancers (stage I: 10.8%, stage II-IV: 89.2%) our study employed mainly stage I colon cancers (90.9%). Thus, it could also be speculated that both miR-17 expression levels and its potential effect on PTEN may be different in early *versus* more advanced colon cancers. The extent to which miR-17 affects PTEN levels should also be taken into consideration, as an *in vitro* study of colon cancer cells has shown that the effect of increased miR-19a on PTEN expression levels is much more pronounced than that of miR-17[57]. Similarly, Olive *et al* found that only miR-19, but not miR-17, affected PTEN expression in murine fibroblast cells (NIH-3T3 cells) and primary B-cell culture[58]. In our study, comparisons of PTEN expression along with miR-19 levels were not conducted.

Xiong *et al* analysed PTEN protein levels along with miR-21 expression by qPCR, a sensitive method that allows a better quantification than ISH, but is unable to provide information about cellular localisation. The assistance of image analysis in the evaluation of miRNA expression could have identified more subtle changes than possible by manual assessment while still obtaining important morphologic knowledge. In addition, considering that *PTEN* is regulated by a number of different genetic and epigenetic mechanisms[55], an more differentiated immunoscore rather than comparing miR-17 and miR-21 expression to complete PTEN loss may have been more rewarding in our study, in that a miRNA probably only affects PTEN protein levels to a certain degree.

In conclusion, the expressions of miR-17 in the epithelial cells and miR-21 in the stroma cells were shown to be up-regulated at an early stage in the N-A-AC sequence, while the number of miR-145 positive pericryptal stromal cells was down-regulated. Thus, the present results support a role of these miRNAs in the oncogenic process of colon cancer and as dynamic biomarkers for early stages of colon cancer development.

## Supporting Information

S1 Fig Immunohistochemical staining of β-catenin in the normal-adenoma-adenocarcinoma sequence. **(A)** Membranous expression and very weak cytoplasmic expression are seen in the normal epithelial cells, while **(B)** moderate cytoplasmic expression and scattered nuclear reaction are found in the adenomatous compartment. **(C)** Nuclear accumulation is seen especially in the tumour budding cells and the invasive front of the adenocarcinoma.
(TIF)

S1 Table miR *in situ* hybridisation experimental details. (DOC)

S2 Table Expression of β-catenin in the normal-adenoma-adenocarcinoma sequence of adenocarcinomas developed in mucosal polyps of the colon. (DOC)

S3 Table Data from the image analysis of miR-17. (XLS)
