# Supplementary figures and images for: microRNA-17 Is the Most Up-Regulated Member of the miR-17-92 Cluster during Early Colon Cancer Evolution
Source: PLoS One. 2015 Oct 14;10(10):e0140503. doi: 10.1371/journal.pone.0140503 (PMC4605595; doi:10.1371/journal.pone.0140503)

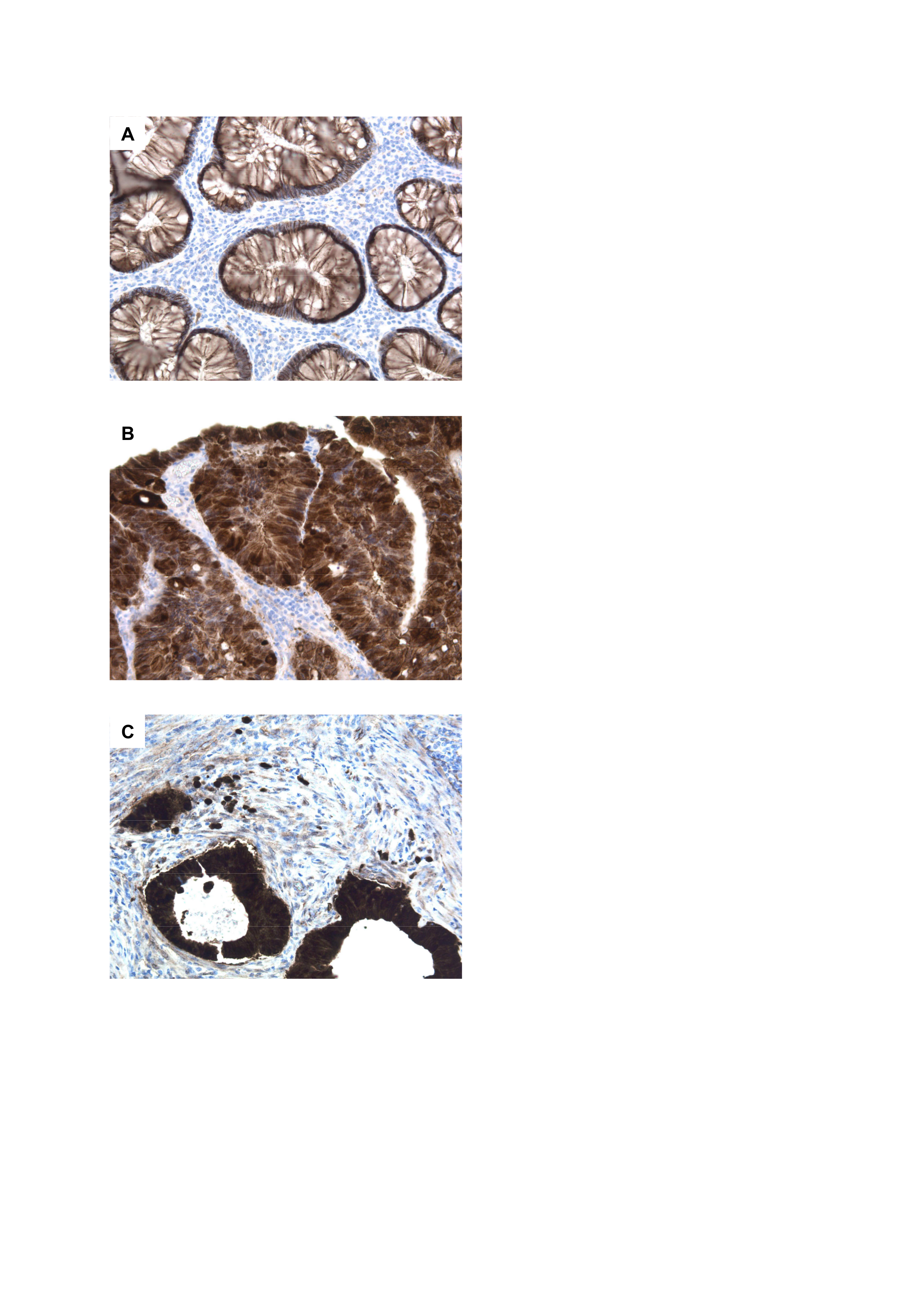

Supplement: S1 Fig — (A) Membranous expression and very weak cytoplasmic expression are seen in the normal epithelial cells, while (B) moderate cytoplasmic expression and scattered nuclear reaction are found in the adenomatous compartment. (C) Nuclear accumulation is seen especially in the tumour budding cells and the invasive front of the adenocarcinoma. (TIF) [file pone.0140503.s001.tif]
