# Supplementary material for: microRNA-17 Is the Most Up-Regulated Member of the miR-17-92 Cluster during Early Colon Cancer Evolution
Source: PLoS One. 2015 Oct 14;10(10):e0140503. doi: 10.1371/journal.pone.0140503 (PMC4605595; doi:10.1371/journal.pone.0140503)
Supplement: S1 Table — (DOC) [file pone.0140503.s002.doc]

S1. microRNA probe sequences, melting and hybridisation temperatures, probe concentration, and substrate incubation time

| Probe name | Sequence | T_m_  (ºC) | T_hyb_  (ºC) | Probe conc (nM) | Substrate incub  (min) |
| --- | --- | --- | --- | --- | --- |
| miR-17 (5p) | TACCTGCACTGTAAGCACTTT | 89 | 55/57^a^ | 40 | 90 |
| miR-18a (5p) | CTATCTGCACTAGATGCACCTTA | 89 | 57 | 40 | 90 |
| miR-19b (3p) | TCAGTTTTGCATGGATTTGCACA | 84 | 57 | 40 | 90 |
| miR-20a (5p) | CTACCTGCACTATAAGCACTTTA | 83 | 57 | 40 | 90 |
| miR-92a (3p) | ACAGGCCGGGACAAGTGCAATA | 84 | 57 | 40 | 90 |
| miR-21 | TCAACATCAGTCTGATAAGCTA | 83 | 57 | 30 | 60 |
| miR-31 (5p) | AGCTATGCCAGCATCTTTGCCT | 88 | 57 | 60 | 90 |
| miR-125b | TCACAAGTTAGGGTCTCAGGGA | 85 | 57 | 40 | 90 |
| miR-126 | CATTATTACTCACGGTACGA | 84 | 57 | 30 | 90 |
| miR-145  miR-200b (3p) | AGGGATTCCTGGGAAAACTGGAC  TCATCATTACCAGGCAGTATTA | 82  85 | 57  55 | 30  40 | 60  90 |
| Scramble | ATGTAACACGTCTATACGCCCA | 86 | 57 | 20 | 90 |

T_m_: melting temperature

T_hyb_: hybridisation temperature

Probe conc: probe concentration

Substrate incub: Substrate incubation time with 4-nitroblue tetrazolium and 5-bromo-4-chloro-3’-Indolylphosphate

^a^55^o^C was used when evaluating miR-17 alone, whereas 57^o^C was used when comparing to the other cluster members.
