# Supplementary material for: microRNA-17 Is the Most Up-Regulated Member of the miR-17-92 Cluster during Early Colon Cancer Evolution
Source: PLoS One. 2015 Oct 14;10(10):e0140503. doi: 10.1371/journal.pone.0140503 (PMC4605595; doi:10.1371/journal.pone.0140503)
Supplement: S2 Table — (DOC) [file pone.0140503.s003.doc]

**S2 Table**.

Expression of β-catenin in the normal-adenoma-adenocarcinoma sequence of adenocarcinomas developed in mucosal polyps of the colon.

|  | **Normal** | **Adenoma** | **Adenocarcinoma** |
| --- | --- | --- | --- |
| **β-catenin, nuclear**  n  Negative  Positive  **β-catenin, cytoplasmic**  n  weak  Moderate  Strong | 24  24 (100%)  0  24  24 (100%)  0  0 | 21^a^  8 (38%)  13 (62%)    21^a^  1 (5%)  12 (57%)  8 (38%) | 21^a^  1 (5%)  20 (95%)  21^a^  0  12 (57%)  9 (43%) |
|  |  |  |  |

**^a^** Data missing due to specimens failing to include all three compartments of normal tissue, adenoma
 and adenocarcinoma.
